# Right-sided infective endocarditis with ventricular septal defect

**DOI:** 10.12669/pjms.40.7.8219

**Published:** 2024-08

**Authors:** Fatina Munawar, Ikram Ahmed

**Affiliations:** 1 Dr. Fatina Munawar, MBBS, Tahir Heart Institute, Fazl-e-Omar Hospital, Chenab Nagar, District Chiniot, Pakistan; 2 Dr. Ikram Ahmed, MBBS, FCPS , Tahir Heart Institute, Fazl-e-Omar Hospital, Chenab Nagar, District Chiniot, Pakistan

**Keywords:** Infective endocarditis, Ventricular septal defect, Antibiotic prophylaxis

## Abstract

Infective endocarditis (IE) affects the endothelium of the heart, with the heart valves most commonly involved. It has been documented that the annual incidence of infective endocarditis is 3-10 per 100,000 patient-years1. However, it can be underestimated since the incidence in developing countries cannot be determined accurately. Here, we present a case of a 37-year-old male who was referred from a local health facility with shortness of breath on presentation; the patient was anuric for one day and initial laboratory investigations showed metabolic acidosis, hyperkalemia, sepsis, and deranged renal function tests. The patient had received a three-week course of intravenous (IV) piperacillin-tazobactam at the previous health facility, being diagnosed as a case of infective endocarditis. An initial transthoracic echocardiogram (TTE) showed vegetation on the pulmonary valve; however, the patient was neither an IV drug abuser nor did he have any history of implantation of intracardiac devices or central venous catheters. There was no recent or remote history of dental or surgical procedures. Due to the acute kidney injury, hemodialysis sessions and IV imipenem were started. As the patient’s hemodynamic profile improved by the fifth day of admission, TTE was repeated, revealing a small ventricular septal defect (VSD). This case report highlights the importance of even small VSD that could potentially lead to right-sided IE. Surgical correction of VSD could prevent such a life-threatening condition.

## INTRODUCTION

The annual incidence of infective endocarditis (IE) is 3-10/100,000 patient-years.^1^ The prevalence of right-sided IE is 5%-10% of all cases of IE owing to intravenous drug abuse as the commonest cause, in addition to the increased use of intra-cardiac devices and central venous catheters, particularly in the past 20 years.^2^ Pulmonary valve IE accounts for only 1.5% to 2% of all cases of IE.^3^ Pulmonary valve endocarditis is a rarer clinical entity as compared to tricuspid valve IE resulting in a lower chance of it being diagnosed in case of clinical suspicion of IE. A finding of pulmonary infiltrates on chest x-ray taken on presentation should guide a clinician towards septic pulmonary embolism: a finding peculiar to pulmonary valve IE.^4^ Right-sided IE is frequently associated with clinical features such as hemoptysis and acute right-heart failure, corresponding to septic pulmonary embolisms and tricuspid regurgitation respectively.^5^

The literature shows that right-sided infective endocarditis may occur in the absence of common risk factors such as intravenous drug abuse, intracardiac devices, and central venous catheters, and a VSD may be suspected as a cause of right-sided infective endocarditis.^6^ The following case report aims to consider VSD in right-sided IE.

## CASE REPORT

A 37-year-old male was referred from a local health facility. On presentation, he was dyspneic and restless. He had a blood pressure of 143/99 mmHg, pulse rate of 124/minute, respiratory rate of 30/minute, oxygen saturation of 95%, and a temperature of 98.6°F. The patient was well-oriented in time, place, and person; he appeared anxious and distressed. Lung examination revealed bilateral, fine inspiratory crepitations up to mid-chest. A Grade-III pansystolic murmur was audible at the left sternal edge radiating to the right. The abdomen was soft and non-tender. Neurological examination was unremarkable. There was no recent or remote history of any dental or surgical procedures. He had received a three-week course of intravenous piperacillin-tazobactam in the previous healthcare facility.

**Fig.1:**
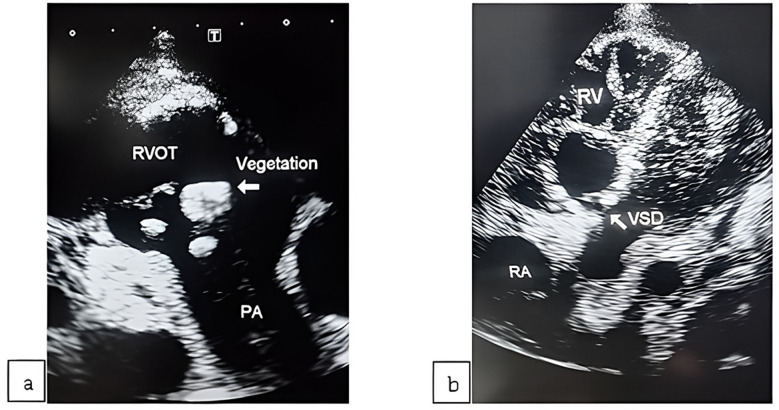
(a) Initial transthoracic echocardiogram. (b) TTE; after five days. RVOT= Right ventricular outflow tract. PA= Pulmonary artery, RA= Right atrium, RV=Right ventricle, VSD= Ventricular septal defect.

An initial complete blood count showed anemia and leukocytosis with neutrophilic predominance. The C-reactive protein (CRP) level was 175mg/L. Mildly raised Troponin-t HS; 49pg/ml (normal < 15pg/ml). Rheumatoid factor was 31.6 IU/ml (cut-off=14 IU/ml). The patient was a known case of Hepatitis-C virus infection and had been taking antiviral medications for the last three months. His liver function tests were normal and there was no liver cirrhosis and ascites. His arterial blood gas analysis showed severe metabolic acidosis (pH=7.02, HCO_3_=6.1mmol/L). The patient was anuric at presentation and his serum creatinine and blood urea levels were 6.9mg/dl and 222mg/dl respectively. The patient was started on hemodialysis. Initial transthoracic echocardiogram revealed vegetations (Fig. 1a: white arrow) in the right ventricular outflow tract (RVOT) and on the pulmonary valve, dilated right-sided chambers, left ventricular (LV) ejection fraction of 50-55%, and a moderate pericardial effusion. Due to tachycardia and respiratory distress, VSD was not identified. Blood cultures were sent, and keeping in mind acute kidney injury and the non-availability of a laboratory facility to monitor drug levels of Vancomycin and Gentamycin, Imipenem was started.^7^ The patient’s clinical condition improved. CRP declined and urine output got better. Five days later, a follow-up transthoracic echocardiogram revealed a small peri-membranous VSD with a left-to-right shunt (Fig.1b: white arrow); pericardial effusion got reduced.

Furthermore, a CT chest and abdomen without contrast was also obtained that showed multifocal areas of consolidation in the lungs, particularly on the left side, and mild pulmonary edema. It also showed circumferential pericardial effusion of up to 2.5 cm in depth. The main pulmonary trunk was enlarged up to 4.0 cm transversely suggesting pulmonary hypertension (Fig.2a).

**Fig.:2:**
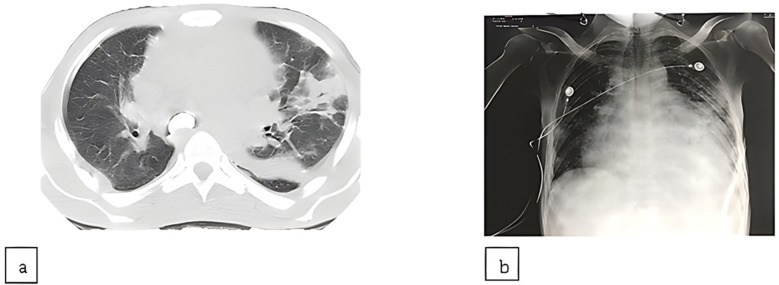
(a) Computed tomography (CT), (b) Chest X-ray chest images without contrast.

The definitive diagnosis of Infective Endocarditis was made using the modified Duke criteria. During the hospital stay, the patient had an episode of fast atrial fibrillation. It was managed by intravenous amiodarone. The patient frequently had episodes of supraventricular tachycardia and he was started on 40mg of verapamil twice daily.

The patient was transfused with one unit of packed RBCs for the anemia (Hemoglobin=8.7g/dl). Blood cultures were negative, possibly due to the three-week antibiotic regimen of tazobactam-piperacillin at the previous hospital. The patient was managed with intravenous imipenem during the hospital stay.

The patient had three sessions of hemodialysis and on the fifth day of admission, his urine output improved and he was off hemodialysis after a Nephrology consult. Cardiothoracic surgical consultation was sought for which medical management was advised.

The left-to-right shunt was hemodynamically stable, and his shortness of breath also got improved. It was decided to continue intravenous imipenem for two more weeks and verapamil twice daily was added to the treatment. A follow-up transthoracic echocardiogram, one month later, showed a reduction in the size of the pulmonary valve vegetation, and absence of pericardial effusion, suggestive of healing.

## DISCUSSION

Right-sided IE usually presents with metastatic manifestations such as septic emboli and acute kidney injury, as in this case. The commonest causative factor for right-sided IE is intravenous (IV) drug abuse. Other risk factors include intra-cardiac devices and central venous catheters.^2^ Pulmonary valve endocarditis constitutes 1.5% - 2% of all cases of IE.^3^ Based on a thorough history and general physical examination, particularly the absence of the telltale signs of IV drug use, the possibility of IV drug abuse and other risk factors was excluded. The absence of these common causative factors and the presence of pansystolic murmur raised the suspicion of VSD but that was not visualized in the first echocardiogram as the patient was in distress. Later, after stabilization, a small peri-membranous VSD with a left-to-right shunt was found on the echocardiogram. Lim WJ et al reported a similar case of pulmonary valve IE that was associated with peri-membranous VSD with a left-to-right shunt.^4^

The 2015 ESC guidelines do not recommend prophylaxis for acyanotic congenital heart disease. The rationale for not recommending prophylaxis before dental procedures is that only an extremely small number of cases of IE might be prevented by antibiotic prophylaxis for dental procedures even if such prophylactic therapy were 100% effective.^5^ Hellwege et al. described tricuspid IE in association with VSD with the left-to-right shunt. The causative organism was Staphylococcus aureus in that case. That case was complicated by the acute renal shutdown and multiple septic pulmonary emboli, like our case. They suggested that congenital VSDs should be considered for antibiotic prophylaxis against endocarditis.^6^ In our case, the patient did not undergo any dental procedure.

There is an increased risk of IE in VSD patients with left-to-right shunt as concluded by Berglund E et al. They found 20-30 times the risk of IE in uncorrected VSD patients with left-to-right shunt as compared to the general population.^8^ This finding could support surgical correction of even small VSDs to prevent IE.

As per the ACC/AHA guideline for the management of adults with congenital heart disease, the VSD-closure-candidates are the patients having left ventricular volume overload and hemodynamically significant shunts (Qp : Qs ≥1.5:1), provided that pulmonary artery (PA) systolic pressure is less than 50% systemic and pulmonary vascular resistance is less than one-third systemic. [COR I, LOE B].^9^

Ventricular septal defects are well-documented factors that predispose the adult population to infective endocarditis. Only a few cases have been reported in the English-language PubMed review that depicts acquired VSD as a clinical sequela of infective endocarditis. One of the cases describes an acquired VSD due to aortic valve infective endocarditis that resulted in rupture and aggravation of aortic regurgitation. Other cases of acquired ventricular septal defects depicted aortic root abscess in association with a mechanical aortic valve and left ventricular outflow tract abscess respectively, that lead to the formation of ventricular septal defects in both the cases.^10^ The case in consideration did not have infective endocarditis due to perivalvular involvement. Hence, it is more likely that the patient had a congenital ventricular septal defect.

## CONCLUSION

This case report highlights the importance of even small VSD that could potentially lead to right-sided IE in addition to other common causes like intravenous drug abuse, intracardiac devices, and central venous catheters. Surgical correction of VSD could prevent such a life-threatening condition.

### Authors Contribution:

**FM:** data collection, manuscript writing, editing, figures, revision.

**IA:** idea, manuscript writing, critical review.

## References

[1] Nishimura RA, Otto CM, Bonow RO, Carabello BA, Erwin JP, Guyton RA (2014). American College of Cardiology/American Heart Association Task Force on Practice. 2014 AHA/ACC guideline for the management of patients with valvular heart disease:a report of the American College of Cardiology/American Heart Association Task Force on Practice Guidelines. J Am Coll Cardiol.

[2] Shmueli H, Thomas F, Flint N, Setia G, Janjic A, Siegel RJ (2020). Right-Sided Infective Endocarditis 2020:Challenges and Updates in Diagnosis and Treatment. J Am Heart Assoc.

[3] Miranda WR, Connolly HM, DeSimone DC, Phillips SD, Wilson WR, Sohail MR (2015). Infective Endocarditis Involving the Pulmonary Valve. Am J Cardiol.

[4] Lim WJ, Kaisbain N, Kim HS (2022). Septic pulmonary emboli in pulmonary valve endocarditis with concurrent ventricular septal defect and coronary artery disease:a case report.

[5] Habib G, Lancellotti P, Antunes MJ, Bongiorni MG, Casalta JP, Del Zotti F (2015). 2015 ESC Guidelines for the management of infective endocarditis:The Task Force for the Management of Infective Endocarditis of the European Society of Cardiology (ESC). Endorsed by:the European Association for Cardio-Thoracic Surgery (EACTS), the European Association of Nuclear Medicine (EANM). Eur Heart J.

[6] Hellwege RS, Gawaz M (2020). Right-sided infective endocarditis in association with a left-to-right shunt complicated by hemoptysis and acute renal failure:a case report. BMC Cardiovascular Disord.

[7] Dickinson G, Rodriguez K, Arcey S, Alea A, Greenman R (1985). Efficacy of imipenem/cilastatin in endocarditis. Am J Med.

[8] Berglund E, Johansson B, Dellborg M, Sörensson P, Christersson C, Nielsen NE (2016). High incidence of infective endocarditis in adults with congenital ventricular septal defect. Heart.

[9] Stout KK, Daniels CJ, Aboulhosn JA, Bozkurt B, Broberg CS, Colman JM (2019). 2018 AHA/ACC Guideline for the Management of Adults With Congenital Heart Disease:Executive Summary:A Report of the American College of Cardiology/American Heart Association Task Force on Clinical Practice Guidelines. J Am Coll Cardiol.

[10] Durden RE, Turek JW, Reinking BE, Bansal M (2018). Acquired ventricular septal defect due to infective endocarditis. Ann Pediat Cardiol.

